# *N*-Phenyl­maleamic acid

**DOI:** 10.1107/S1600536809014627

**Published:** 2009-04-25

**Authors:** Kong Mun Lo, Seik Weng Ng

**Affiliations:** a Department of Chemistry, University of Malaya, 50603 Kuala Lumpur, Malaysia

## Abstract

The two independent mol­ecules in the title compound (systematic name: 4-amino-4-oxobut-2-enoic acid), C_10_H_9_NO_3_, are both essentially planar (r.m.s. deviations = 0.05 and 0.06 Å). In both mol­ecules, the –OH group forms an intra­molecular hydrogen bond to the amide O atom. Adjacent mol­ecules are linked by N—H⋯O hydrogen bonds into a flat ribbon that runs along the *a* axis of the monoclinic unit cell.

## Related literature

For the crystal structures of other substituted *N*-(phen­yl)maleamic acids, see, for example: Gonzalez-Rodriguez *et al.* (1986 ▶); Home *et al.* (1991 ▶); Lynch & McClenaghan (2002 ▶); Parvez *et al.* (2004*a* ▶,*b* ▶); Prasad *et al.* (2002*a* ▶,*b* ▶); Santos-Sánchez *et al.* (2007 ▶); Wardell *et al.* (2005 ▶).
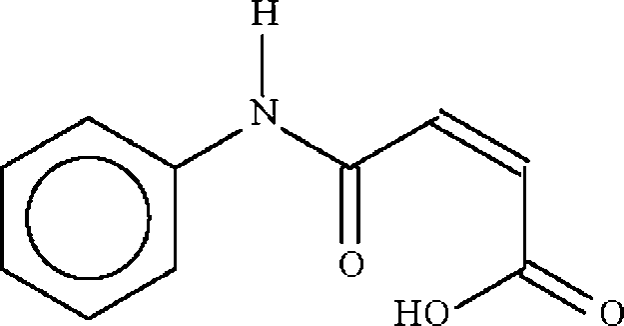


## Experimental

### 

#### Crystal data

C_10_H_9_NO_3_
*M* *_r_* = 191.18
Monoclinic, 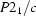

*a* = 12.7505 (4) Å
*b* = 10.5849 (5) Å
*c* = 14.1918 (6) Å
β = 116.299 (3)°
*V* = 1717.1 (1) Å^3^
*Z* = 8
Mo *K*α radiation
μ = 0.11 mm^−1^
*T* = 100 K
0.24 × 0.06 × 0.06 mm

#### Data collection

Bruker SMART APEX diffractometer
Absorption correction: none
11493 measured reflections
3925 independent reflections
2256 reflections with *I* > 2σ(*I*)
*R*_int_ = 0.062

#### Refinement

*R*[*F* ^2^ > 2σ(*F* ^2^)] = 0.068
*wR*(*F* ^2^) = 0.208
*S* = 1.02
3925 reflections
269 parameters
4 restraints
H atoms treated by a mixture of independent and constrained refinement
Δρ_max_ = 0.96 e Å^−3^
Δρ_min_ = −0.36 e Å^−3^

### 

Data collection: *APEX2* (Bruker, 2008 ▶); cell refinement: *SAINT* (Bruker, 2008 ▶); data reduction: *SAINT*; program(s) used to solve structure: *SHELXS97* (Sheldrick, 2008 ▶); program(s) used to refine structure: *SHELXL97* (Sheldrick, 2008 ▶); molecular graphics: *X-SEED* (Barbour, 2001 ▶); software used to prepare material for publication: *publCIF* (Westrip, 2009 ▶).

## Supplementary Material

Crystal structure: contains datablocks global, I. DOI: 10.1107/S1600536809014627/tk2436sup1.cif

Structure factors: contains datablocks I. DOI: 10.1107/S1600536809014627/tk2436Isup2.hkl

Additional supplementary materials:  crystallographic information; 3D view; checkCIF report

## Figures and Tables

**Table 1:** Hydrogen-bond geometry (Å, °)

*D*—H⋯*A*	*D*—H	H⋯*A*	*D*⋯*A*	*D*—H⋯*A*
O2—H2o⋯O3	0.85 (1)	1.63 (1)	2.475 (3)	172 (4)
O5—H5o⋯O6	0.86 (1)	1.65 (1)	2.496 (3)	170 (3)
N1—H1n⋯O4	0.88 (1)	2.00 (1)	2.864 (3)	166 (3)
N2—H2n⋯O1^i^	0.89 (1)	1.99 (1)	2.859 (3)	167 (3)

Symmetry code: (i) 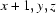.

**Fig. 1.**
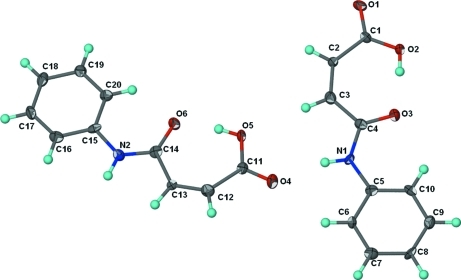
Thermal ellipsoid plot (Barbour, 2001) of the two independent molecules of C10H9NO3 at the 70% probability level. Hydrogen atoms are drawn as spheres of arbitrary radius.

## supplementary crystallographic information

### Experimental

Maleic anhydride (1 g, 1 mmol) and aniline (1 ml, 1 mmol) was heated in toluene (50 ml) for 1 h. The solution was set aside for the formation of crystals.

### Refinement

Carbon-bound H-atoms were placed in calculated positions (C–H 0.95 Å) and were included in the refinement in the riding model approximation, with *U*(H) set to 1.2*U*(C). The oxygen- and nitrogen-bound hydrogen atoms were located in a difference Fourier map, and were refined with distance restraints of O–H 0.84±0.01 Å and N–H 0.88±0.01 Å; the temperature factors were refined.

### Crystal data

**Table:**

C_10_H_9_NO_3_	*F*(000) = 800
*M**_r_* = 191.18	*D*_x_ = 1.479 Mg m^−^^3^
Monoclinic, *P*2_1_/*c*	Mo *K*α radiation, λ = 0.71073 Å
Hall symbol: -P 2ybc	Cell parameters from 1202 reflections
*a* = 12.7505 (4) Å	θ = 2.6–27.4°
*b* = 10.5849 (5) Å	µ = 0.11 mm^−^^1^
*c* = 14.1918 (6) Å	*T* = 100 K
β = 116.299 (3)°	Prism, colorless
*V* = 1717.1 (1) Å^3^	0.24 × 0.06 × 0.06 mm
*Z* = 8	

### Data collection

**Table:**

Bruker SMART APEX diffractometer	2256 reflections with *I* > 2σ(*I*)
Radiation source: fine-focus sealed tube	*R*_int_ = 0.062
graphite	θ_max_ = 27.5°, θ_min_ = 1.8°
ω scans	*h* = −16→16
11493 measured reflections	*k* = −13→13
3925 independent reflections	*l* = −14→18

### Refinement

**Table:**

Refinement on *F*^2^	Primary atom site location: structure-invariant direct methods
Least-squares matrix: full	Secondary atom site location: difference Fourier map
*R*[*F*^2^ > 2σ(*F*^2^)] = 0.068	Hydrogen site location: inferred from neighbouring sites
*wR*(*F*^2^) = 0.208	H atoms treated by a mixture of independent and constrained refinement
*S* = 1.02	*w* = 1/[σ^2^(*F*_o_^2^) + (0.1139*P*)^2^] where *P* = (*F*_o_^2^ + 2*F*_c_^2^)/3
3925 reflections	(Δ/σ)_max_ = 0.001
269 parameters	Δρ_max_ = 0.96 e Å^−^^3^
4 restraints	Δρ_min_ = −0.36 e Å^−^^3^

### Fractional atomic coordinates and isotropic or equivalent isotropic displacement parameters (Å^2^)

**Table:**

	*x*	*y*	*z*	*U*_iso_*/*U*_eq_	
O1	0.41604 (16)	0.86211 (19)	0.61750 (16)	0.0173 (5)	
O2	0.41080 (17)	0.6545 (2)	0.62972 (17)	0.0167 (5)	
H2O	0.449 (3)	0.590 (3)	0.627 (3)	0.058 (14)*	
O3	0.53761 (17)	0.4779 (2)	0.62899 (16)	0.0187 (5)	
O4	0.91395 (17)	0.5468 (2)	0.60999 (17)	0.0216 (5)	
O5	0.91031 (17)	0.75293 (19)	0.62870 (17)	0.0164 (5)	
H5O	0.946 (3)	0.820 (2)	0.625 (3)	0.028 (10)*	
O6	1.03470 (16)	0.93320 (19)	0.62520 (16)	0.0187 (5)	
N1	0.7136 (2)	0.4490 (2)	0.62704 (18)	0.0122 (5)	
H1N	0.771 (2)	0.492 (3)	0.623 (2)	0.019 (8)*	
N2	1.2093 (2)	0.9624 (2)	0.62037 (19)	0.0149 (6)	
H2N	1.2657 (19)	0.923 (3)	0.611 (2)	0.020 (8)*	
C1	0.4591 (2)	0.7584 (3)	0.6178 (2)	0.0123 (6)	
C2	0.5691 (2)	0.7538 (3)	0.6046 (2)	0.0109 (6)	
H2	0.5933	0.8336	0.5906	0.013*	
C3	0.6396 (2)	0.6584 (3)	0.6087 (2)	0.0157 (6)	
H3	0.7079	0.6811	0.6018	0.019*	
C4	0.6250 (2)	0.5224 (3)	0.6227 (2)	0.0135 (6)	
C5	0.7229 (2)	0.3155 (3)	0.6348 (2)	0.0130 (6)	
C6	0.8169 (2)	0.2612 (3)	0.6232 (2)	0.0118 (6)	
H6	0.8710	0.3139	0.6125	0.014*	
C7	0.8311 (2)	0.1321 (3)	0.6272 (2)	0.0164 (6)	
H7	0.8945	0.0957	0.6188	0.020*	
C8	0.7521 (2)	0.0547 (3)	0.6436 (2)	0.0149 (6)	
H8	0.7609	−0.0345	0.6450	0.018*	
C9	0.6615 (2)	0.1075 (3)	0.6576 (2)	0.0171 (6)	
H9	0.6086	0.0544	0.6697	0.020*	
C10	0.6463 (2)	0.2388 (3)	0.6541 (2)	0.0136 (6)	
H10	0.5843	0.2749	0.6648	0.016*	
C11	0.9577 (2)	0.6509 (3)	0.6128 (2)	0.0145 (6)	
C12	1.0671 (2)	0.6566 (3)	0.5988 (2)	0.0171 (6)	
H12	1.0910	0.5773	0.5836	0.021*	
C13	1.1380 (2)	0.7531 (3)	0.6038 (2)	0.0095 (6)	
H13	1.2070	0.7313	0.5977	0.011*	
C14	1.1215 (2)	0.8896 (3)	0.6177 (2)	0.0140 (6)	
C15	1.2203 (2)	1.0966 (3)	0.6289 (2)	0.0130 (6)	
C16	1.3130 (2)	1.1490 (3)	0.6154 (2)	0.0169 (7)	
H16	1.3645	1.0961	0.6012	0.020*	
C17	1.3298 (2)	1.2781 (3)	0.6228 (2)	0.0163 (6)	
H17	1.3934	1.3136	0.6143	0.020*	
C18	1.2542 (2)	1.3569 (3)	0.6427 (2)	0.0165 (6)	
H18	1.2659	1.4458	0.6471	0.020*	
C19	1.1619 (2)	1.3044 (3)	0.6560 (2)	0.0147 (6)	
H19	1.1097	1.3576	0.6689	0.018*	
C20	1.1457 (2)	1.1746 (3)	0.6504 (2)	0.0147 (6)	
H20	1.0835	1.1388	0.6612	0.018*	

### Atomic displacement parameters (Å^2^)

**Table:**

	*U*^11^	*U*^22^	*U*^33^	*U*^12^	*U*^13^	*U*^23^
O1	0.0154 (10)	0.0149 (12)	0.0241 (12)	0.0036 (8)	0.0111 (9)	−0.0025 (9)
O2	0.0143 (10)	0.0118 (12)	0.0283 (12)	0.0004 (9)	0.0133 (9)	0.0026 (9)
O3	0.0165 (10)	0.0144 (12)	0.0298 (12)	−0.0003 (8)	0.0145 (9)	0.0015 (9)
O4	0.0195 (10)	0.0149 (12)	0.0335 (13)	−0.0042 (9)	0.0145 (10)	−0.0003 (9)
O5	0.0150 (10)	0.0108 (12)	0.0280 (13)	−0.0026 (8)	0.0138 (9)	0.0000 (9)
O6	0.0176 (10)	0.0126 (12)	0.0322 (13)	−0.0003 (9)	0.0166 (9)	−0.0029 (9)
N1	0.0159 (12)	0.0072 (13)	0.0159 (12)	−0.0001 (10)	0.0091 (10)	0.0012 (10)
N2	0.0145 (12)	0.0157 (14)	0.0175 (13)	0.0013 (10)	0.0097 (10)	0.0010 (10)
C1	0.0106 (13)	0.0144 (16)	0.0125 (15)	0.0004 (11)	0.0057 (11)	−0.0015 (11)
C2	0.0111 (13)	0.0083 (15)	0.0136 (14)	−0.0008 (10)	0.0057 (11)	−0.0022 (11)
C3	0.0166 (13)	0.0157 (17)	0.0185 (15)	−0.0007 (12)	0.0112 (12)	−0.0009 (12)
C4	0.0135 (13)	0.0150 (16)	0.0128 (14)	0.0017 (11)	0.0067 (11)	−0.0012 (11)
C5	0.0149 (13)	0.0136 (15)	0.0106 (14)	0.0022 (12)	0.0056 (11)	0.0007 (12)
C6	0.0127 (13)	0.0119 (15)	0.0128 (14)	−0.0023 (11)	0.0075 (11)	0.0004 (11)
C7	0.0161 (13)	0.0162 (17)	0.0165 (15)	0.0017 (12)	0.0068 (12)	−0.0009 (12)
C8	0.0231 (14)	0.0050 (14)	0.0177 (15)	−0.0012 (11)	0.0101 (12)	−0.0002 (11)
C9	0.0166 (13)	0.0175 (17)	0.0171 (15)	−0.0020 (12)	0.0075 (12)	0.0044 (12)
C10	0.0094 (12)	0.0195 (17)	0.0122 (15)	0.0029 (11)	0.0049 (11)	0.0005 (12)
C11	0.0151 (13)	0.0139 (16)	0.0159 (15)	0.0000 (12)	0.0082 (12)	0.0018 (12)
C12	0.0197 (14)	0.0182 (17)	0.0175 (15)	0.0032 (12)	0.0119 (12)	0.0005 (12)
C13	0.0114 (12)	0.0100 (14)	0.0075 (13)	0.0000 (10)	0.0044 (10)	−0.0009 (10)
C14	0.0176 (13)	0.0158 (16)	0.0091 (14)	0.0013 (12)	0.0064 (11)	0.0009 (11)
C15	0.0176 (13)	0.0107 (15)	0.0084 (13)	0.0042 (11)	0.0037 (11)	0.0025 (11)
C16	0.0171 (14)	0.0180 (17)	0.0167 (15)	0.0033 (12)	0.0083 (12)	0.0000 (12)
C17	0.0105 (13)	0.0211 (17)	0.0164 (15)	−0.0028 (12)	0.0051 (11)	0.0027 (13)
C18	0.0173 (14)	0.0139 (16)	0.0154 (15)	−0.0052 (12)	0.0046 (12)	−0.0002 (12)
C19	0.0135 (13)	0.0141 (16)	0.0155 (15)	−0.0006 (12)	0.0057 (11)	−0.0011 (12)
C20	0.0159 (13)	0.0149 (15)	0.0136 (15)	0.0020 (12)	0.0069 (11)	0.0005 (12)

### Geometric parameters (Å, °)

**Table:**

O1—C1	1.227 (3)	C7—C8	1.395 (4)
O2—C1	1.307 (3)	C7—H7	0.9500
O2—H2O	0.846 (10)	C8—C9	1.375 (4)
O3—C4	1.249 (3)	C8—H8	0.9500
O4—C11	1.227 (3)	C9—C10	1.401 (4)
O5—C11	1.305 (3)	C9—H9	0.9500
O5—H5O	0.857 (10)	C10—H10	0.9500
O6—C14	1.247 (3)	C11—C12	1.495 (4)
N1—C4	1.350 (3)	C12—C13	1.344 (4)
N1—C5	1.419 (4)	C12—H12	0.9500
N1—H1N	0.879 (10)	C13—C14	1.486 (4)
N2—C14	1.345 (4)	C13—H13	0.9500
N2—C15	1.427 (4)	C15—C20	1.391 (4)
N2—H2N	0.886 (10)	C15—C16	1.394 (4)
C1—C2	1.496 (4)	C16—C17	1.379 (4)
C2—C3	1.335 (4)	C16—H16	0.9500
C2—H2	0.9500	C17—C18	1.394 (4)
C3—C4	1.476 (4)	C17—H17	0.9500
C3—H3	0.9500	C18—C19	1.389 (4)
C5—C10	1.387 (4)	C18—H18	0.9500
C5—C6	1.403 (4)	C19—C20	1.387 (4)
C6—C7	1.377 (4)	C19—H19	0.9500
C6—H6	0.9500	C20—H20	0.9500
			
C1—O2—H2O	111 (3)	C10—C9—H9	119.7
C11—O5—H5O	112 (2)	C5—C10—C9	119.3 (3)
C4—N1—C5	128.0 (2)	C5—C10—H10	120.3
C4—N1—H1N	114 (2)	C9—C10—H10	120.3
C5—N1—H1N	118 (2)	O4—C11—O5	120.7 (2)
C14—N2—C15	128.4 (2)	O4—C11—C12	118.0 (3)
C14—N2—H2N	117 (2)	O5—C11—C12	121.3 (3)
C15—N2—H2N	115 (2)	C13—C12—C11	132.0 (3)
O1—C1—O2	121.2 (2)	C13—C12—H12	114.0
O1—C1—C2	118.1 (2)	C11—C12—H12	114.0
O2—C1—C2	120.7 (2)	C12—C13—C14	127.9 (2)
C3—C2—C1	132.0 (3)	C12—C13—H13	116.0
C3—C2—H2	114.0	C14—C13—H13	116.0
C1—C2—H2	114.0	O6—C14—N2	123.0 (3)
C2—C3—C4	128.4 (3)	O6—C14—C13	123.6 (3)
C2—C3—H3	115.8	N2—C14—C13	113.4 (2)
C4—C3—H3	115.8	C20—C15—C16	119.9 (3)
O3—C4—N1	122.3 (3)	C20—C15—N2	123.9 (3)
O3—C4—C3	123.1 (3)	C16—C15—N2	116.2 (2)
N1—C4—C3	114.5 (2)	C17—C16—C15	119.7 (3)
C10—C5—C6	119.8 (3)	C17—C16—H16	120.1
C10—C5—N1	123.8 (2)	C15—C16—H16	120.1
C6—C5—N1	116.4 (2)	C16—C17—C18	120.7 (3)
C7—C6—C5	120.3 (3)	C16—C17—H17	119.6
C7—C6—H6	119.8	C18—C17—H17	119.6
C5—C6—H6	119.8	C19—C18—C17	119.4 (3)
C6—C7—C8	119.9 (3)	C19—C18—H18	120.3
C6—C7—H7	120.0	C17—C18—H18	120.3
C8—C7—H7	120.0	C20—C19—C18	120.2 (3)
C9—C8—C7	120.0 (3)	C20—C19—H19	119.9
C9—C8—H8	120.0	C18—C19—H19	119.9
C7—C8—H8	120.0	C19—C20—C15	120.1 (3)
C8—C9—C10	120.6 (3)	C19—C20—H20	120.0
C8—C9—H9	119.7	C15—C20—H20	120.0
			
O1—C1—C2—C3	174.8 (3)	O4—C11—C12—C13	−175.7 (3)
O2—C1—C2—C3	−5.1 (5)	O5—C11—C12—C13	3.6 (5)
C1—C2—C3—C4	3.8 (5)	C11—C12—C13—C14	−5.2 (5)
C5—N1—C4—O3	2.6 (4)	C15—N2—C14—O6	−1.8 (5)
C5—N1—C4—C3	−176.7 (3)	C15—N2—C14—C13	177.6 (2)
C2—C3—C4—O3	2.7 (5)	C12—C13—C14—O6	−1.1 (5)
C2—C3—C4—N1	−178.0 (3)	C12—C13—C14—N2	179.4 (3)
C4—N1—C5—C10	−9.5 (4)	C14—N2—C15—C20	9.1 (4)
C4—N1—C5—C6	171.4 (3)	C14—N2—C15—C16	−171.5 (3)
C10—C5—C6—C7	2.3 (4)	C20—C15—C16—C17	−0.4 (4)
N1—C5—C6—C7	−178.5 (2)	N2—C15—C16—C17	−179.8 (2)
C5—C6—C7—C8	−0.4 (4)	C15—C16—C17—C18	−0.6 (4)
C6—C7—C8—C9	−1.2 (4)	C16—C17—C18—C19	0.5 (4)
C7—C8—C9—C10	0.9 (4)	C17—C18—C19—C20	0.5 (4)
C6—C5—C10—C9	−2.5 (4)	C18—C19—C20—C15	−1.5 (4)
N1—C5—C10—C9	178.4 (3)	C16—C15—C20—C19	1.5 (4)
C8—C9—C10—C5	0.9 (4)	N2—C15—C20—C19	−179.2 (2)

### Hydrogen-bond geometry (Å, °)

**Table:**

*D*—H···*A*	*D*—H	H···*A*	*D*···*A*	*D*—H···*A*
O2—H2o···O3	0.85 (1)	1.63 (1)	2.475 (3)	172 (4)
O5—H5o···O6	0.86 (1)	1.65 (1)	2.496 (3)	170 (3)
N1—H1n···O4	0.88 (1)	2.00 (1)	2.864 (3)	166 (3)
N2—H2n···O1^i^	0.89 (1)	1.99 (1)	2.859 (3)	167 (3)

Symmetry codes: (i) *x*+1, *y*, *z*.
